# Cardiac computed tomography angiography‐derived analysis of left atrial appendage morphology and left atrial dimensions for the prediction of atrial fibrillation recurrence after pulmonary vein isolation

**DOI:** 10.1002/clc.23743

**Published:** 2021-10-14

**Authors:** Florian Straube, Janis Pongratz, Stefan Hartl, Benedikt Brueck, Christian Tesche, Ullrich Ebersberger, Thomas Helmberger, Alexander Crispin, Michael Wankerl, Uwe Dorwarth, Ellen Hoffmann

**Affiliations:** ^1^ Heart Center Munich‐Bogenhausen, Department of Cardiology and Internal Intensive Care Medicine, Munich Clinic Bogenhausen Academic Teaching Hospital of the Technical University Munich Munich Germany; ^2^ Department of Radiology, Neuroradiology and Nuclear Medicine, Munich Clinic Bogenhausen Academic Teaching Hospital of the Technical University Munich Munich Germany; ^3^ Institute for Medical Information Processing, Biometry and Epidemiology Ludwig‐Maximilians‐University Munich Germany; ^4^ Department of Cardiology St. Johannes Hospital Dortmund Dortmund Germany; ^5^ Faculty of Medicine and the University Hospital, Dept. of Cardiology Ludwig‐Maximilians‐University Munich Germany; ^6^ KMN Kardiologie Muenchen Nord Munich Germany; ^7^ Internal Medicine and Cardiology Kardiologie Erkelenz Erkelenz Germany

**Keywords:** atrial fibrillation, cryoballoon ablation, left atrial appendage, morphology, pulmonary vein isolation

## Abstract

**Background:**

Left atrial appendage (LAA) is a potential source of atrial fibrillation (AF) triggers.

**Hypothesis:**

LAA morphology and dimensions are associated with AF recurrence after pulmonary vein isolation (PVI).

**Methods:**

From cardiac computed tomography angiography (CCTA), left atrial (LA), pulmonary vein (PV), and LAA anatomy were assessed in cryoballoon ablation (CBA) patients.

**Results:**

Among 1103 patients undergoing second‐generation CBA, 725 (65.7%) received CCTA with 473 (42.9%) qualifying for detailed LAA analysis (66.3 ± 9.5 years). Symptomatic AF reoccurred in 166 (35.1%) patients during a median follow‐up of 19 months. Independent predictors of recurrence were LA volume, female sex, and mitral regurgitation ≥°II. LAA volume and AF‐type were dependent predictors of recurrence due to their strong correlations with LA volume. LA volumes ≥122.7 ml (sensitivity 0.53, specificity 0.69, area under the curve [AUC] 0.63) and LAA volumes ≥11.25 ml (sensitivity 0.39, specificity 0.79, AUC 0.59) were associated with recurrence. LA volume was significantly smaller in females. LAA volumes showed no sex‐specific difference. LAA morphology, classified as windsock (51.4%), chicken‐wing (20.7%), cactus (12.5%), and cauliflower‐type (15.2%), did not predict successful PVI (log‐rank; *p* = 0.596).

**Conclusions:**

LAA volume was strongly correlated to LA volume and was a dependent predictor of recurrence after CBA. Main independent predictors were LA volume, female sex, and mitral regurgitation ≥°II. Gender differences in LA volumes were observed. Individual LAA morphology was not associated with AF recurrence after cryo‐PVI. Our results indicate that preprocedural CCTA might be a useful imaging modality to evaluate ablation strategies for patients with recurrences despite successful PVI.

## 1 INTRODUCTION

Left atrial appendage (LAA) morphology can be determined by cardiac computed tomography angiography (CCTA). However, the role of the LAA in the initiation and perpetuation of AF has to be defined further. Pulmonary vein electrical isolation (PVI) is the standard strategy at the initial ablation procedure for the treatment of paroxysmal and persistent atrial fibrillation (AF).^1^ Despite continuing technical improvements,^2^ a significant proportion of patients have recurrence after the first ablation procedure.^3^ Several mechanisms causing recurrence have been identified. Electrical reconnection of the PVs is the main mechanism, and gap closure in the second procedure remains the standard of care.^1^, ^4^, ^5^ With the introduction of next generation ablation catheters and smart ablation protocols, the majority of PVs show durable isolation.^4^, ^6^, ^7^ Additional lesions beyond PV isolation (PVI) (e.g., empirical lines, complex fractionated electrograms, ganglionated plexi, triggers) have been studied, but until now, these measures have not demonstrated additional benefit as compared to PVI alone in the initial ablation procedure.^8^ Clinical predictors of AF recurrence after catheter ablation are LA size, AF type, female sex, and in‐hospital AF relapse, as well as comorbidities such as impaired cardiac and renal function.^9^, ^10^, ^11^ LAA itself might also be a source of extra‐PV AF triggers^12^ or might serve as the substrate of perpetuating AF, though it is unknown whether the LAA anatomy correlates with the recurrence rate after PVI. LAA is a complex anatomical structure with substantial variation in size and morphology; it is not possible to determine LAA morphology or LAA size through transthoracic echocardiography (TTE). However, CCTA is the best modality to determine LAA morphology and atrial dimensions.^13^ Other benefits from preprocedural cardiac computed tomography angiography (CCTA) include ruling out possible thrombi, detecting underlying coronary artery disease (CAD), and gaining advanced information of individual PV anatomy.^1^, ^14^

The present study sought to investigate whether LAA morphology and detailed measurement of a variety of LAA and LA parameters, determined by CCTA, can predict atrial arrhythmia recurrence after initial cryoballoon PVI in symptomatic AF patients.

## 2 METHODS

### 2.1 Study design

This prospective single‐centre registry study enrolled consecutive patients undergoing second‐generation CBA between May 2012 and September 2016. Informed consent was obtained from all patients. The study was approved by the regional ethics review board and was conducted in accordance with the Declaration of Helsinki.

### 2.2 Study participants

Consecutive symptomatic patients scheduled for the initial AF ablation procedure aiming at PV isolation were treated with the second‐generation cryoballoon (Arctic Front Advance™, Medtronic Inc., MN, USA) and were prospectively enrolled into the institutional observational registry. If a recent preprocedural CCTA of sufficient quality to assess the LAA anatomy was available, the patient was considered for the present blinded analysis. The clinical indications for CCTA were, firstly, exclusion of CAD, and secondly, determination of LA and PV anatomy prior to the CBA. No patient was excluded from cryoballoon ablation based on variations in LA, PV, LAA anatomy, or LA volume, as determined by CCTA. None of the consecutive patients were treated by means of point‐by‐point radiofrequency (RF) ablation for PVI. Standard exclusion criteria for AF ablation were applied.^15^ Baseline characteristics were collected prospectively.

### 2.3 Objectives and endpoints

The primary objective of the study was to evaluate the impact of LAA anatomy and morphology on the recurrence of AF after CBA. The secondary objective was the general determination of possible independent clinical risk factors for AF recurrence.

### 2.4 Preprocedural investigations

Prior to the ablation procedure, all patients underwent transthoracic and transesophageal echocardiography to exclude possible LA thrombus formation. Additionally, individual left atrial anatomy was revealed using a 64‐slice CT scanner (Brilliance 64, Philips Medical Systems, Cleveland, OH, USA) with retrospective electrocardiography (ECG) gating and 3D reconstruction prior to the procedure. Scanning was performed at 120 kVp, with an effective tube current of 600 mAs. The slice collimation was 64 × 0.625 mm, with a gantry rotation time of 0.4 s and a pitch of 0.2. Images were reconstructed at 0.9 mm slice thickness at increments of 0.45 mm. Contrast enhancement with 80 ml of contrast agent (Imeron 400 MCT, Iomeprol 81.65 g/100 ml, Bracco, Konstanz, Germany) was injected at a flow rate of 5 ml/s and followed by a 50‐ml saline flush. A blinded observer (JP) analyzed, segmented, and measured each CCTA image visually and quantitatively with regard to the defined parameters and the morphological classification of the LAA using EnSite Precision™ (Abbott Medical GmbH, Eschborn, Germany). Multiplan volume‐rendered post‐processing was used to acquire a 3D perspective. After anatomical segmentation into PV, LA, and LAA, all 2‐dimensional (2D) and 3D measurements were conducted.

### 2.5 Data acquisition, management and quality control

The main 2D parameters included the maximal length, height, and depth of the LAA. The volume of the LAA and LA was computed automatically after anatomical segmentation. Major parameters analyzed three‐dimensionally were the LAA length, roof top and bottom lines, distance from the mitral valve annulus to the middle of the LA roof, depth of the LA, and septum orifice distance (see Figure S3). Major subsequent computed parameters were the ellipsoidal area of the LAA ostium, perimeter calculated by Ramanujan's formula, and trapezoid area of the posterior wall calculated by the roof top line, and roof and bottom lines and the distance between those lines. Based on the criteria established by Wang et al. and Kimura et al., the LAA was classified into one of four types: windsock, chicken‐wing, cactus, and cauliflower.^16^, ^17^ After initial classification, there was a reassessment of all images among a team of four physicians for objective validation. CBA was performed as previously described^15^, ^18^ (Appendix).

All ablation procedures were performed by experienced interventional electrophysiologists (EH, UD, FS, MW). Following the intervention, all patients were monitored with continuous ECG for at least 24–48 h. TTE was performed to exclude pericardial effusion. In the event of symptoms, additional ECG and Holter studies were continued for up to 7 days. Holter recordings after 1, 3, 6, and at least 12 months were organized to screen for symptomatic or asymptomatic atrial arrhythmias. Follow‐up was also ensured in cooperation with referring physicians, and detailed questionnaires (15 questions) were administered for each case via mail and telephone calls. If there was any suspicion of recurrence, the referring physician was contacted to validate the diagnosis. Only recurrences outside of the 90‐day blanking period were categorized as failures.

### 2.6 Statistical analysis

Statistical analysis was performed using Microsoft Excel 2016 (Microsoft Corp., Redmond, WA, USA) and SPSS version 25 (IBM Corp., Armonk, NY, USA). Categorical variables are reported as numbers and percentages. In accordance with the Shapiro–Wilk test, continuous variables are expressed as means with standard deviations (SD) or as medians with quartiles. To assess the relationship between LAA parameters and the recurrence of AF after CBA, univariate Cox regressions and Kaplan–Meier plots with log‐rank tests were used. To avoid problems regarding multicollinearity in multivariate models Pearson's and Spearman's correlation coefficients were computed to identify intervariable relationships.Main independent risk factors were determined using a stepwise multivariate Cox regression model with bidirectional elimination including only parameters of highest univariate significance. Parameters highly correlated with these main features were eliminated. Receiver operating characteristic (ROC) analysis with the corresponding area under the curve (AUC) was performed to determine the specific cut‐off values. Statistical significance was defined as *p* ≤ 0.05. All data were analyzed using the SPSS version 25.

## 3 RESULTS

### 3.1 Study population and procedural results

From May 2012 to September 2016, 1103 patients underwent PVI for symptomatic paroxysmal or persistent AF. CCTA was performed in 725 (65.7%) patients, and 473 (42.9%) patients had sufficient image quality for LAA measurements. Figure S1 represents a flow diagram of the study. The mean age of patients who underwent PVI was 66.2 ± 9.5 years, of whom, 189 (40%) were females. The majority of patients (58.6%) had symptomatic paroxysmal AF. Table 1 illustrates the baseline characteristics of the study population in terms of recurrence and non‐recurrence.

**TABLE 1:** Baseline characteristics

	All patients		With recurrence		Without recurrence		*p*‐value
	Value	Analyzed	Value	Analyzed	Value	Analyzed	
Age, years	66.25 ± 9.47	473	67.36 ± 9.28	166	65.65 ± 9.53	307	0.05
Females (%)	189 (40.0)	473	74 (15.6)	166	115 (24.3)	307	0.08
Persistent AF (%)	196 (41.4)	473	82 (17.3)	166	114 (24.1)	307	0.006
Mitral regurgitation ≥ °II	18 (3.8)	473	10 (2.1)	166	8 (1.7)	307	0.005
Valve disease ≥ °II	25 (5.3)	473	13 (2.7)	166	12 (2.5)	307	0.007
LA diameter^a^ mm	43 [40; 47]	440	45 [41; 50]	159	42 [39; 47]	281	0.002
Ejection fraction^a^ (%)	56.88 ± 6.1	459	56.39 ± 5.64	162	57.14 ± 6.32	297	0.72
Hypertension	309 (66.2)	467	112 (24.0)	164	197 (42.1)	303	0.51
Hypertensive heart disease^a^	103 (21.8)	467	43 (9.2)	164	60 (12.8)	303	0.10
Cardiomyopathy	16 (3.4)	466	3 (0.6)	163	13 (2.8)	303	0.2
Prior myocardial infarction	8 (1.7)	465	3 (0.6)	162	5 (1.1)	303	0.98
Structural heart disease	170 (35.9)	473	69 (14.6)	166	101 (21.3)	307	0.042
Overweight (BMI > 25)	233 (61.6)	378	80 (21.2)	137	153 (40.4)	241	0.21
Obesity (BMI > 30)	67 (17.7)	378	21 (5.6)	137	46 (12.2)	241	0.21
Obesity °II/III (BMI > 35)	20 (5.3)	378	9 (2.4)	137	11 (3.0)	241	0.64
Left common ostium^b^	101 (21.4)	473	37 (7.8)	166	64 (13.5)	307	0.81
Accessory veins^b^	83 (17.5)	473	37 (7.8)	166	46 (9.7)	307	0.053

*Note*: *n* (%), mean ± SD, or median (IQR).

Abbreviations: AF, atrial fibrillation; BMI, body mass index; IQR, interquartile range; LA, left atrium; SD, standard deviation.

^a^ Determined by transthoracic echocardiography.

^b^ Determined by contrast enhanced cardiac computer tomography.

All patients underwent CBA, and all PVs were successfully isolated with the cryoballoon. No additional RF or Cryo‐Tip touch‐up ablations were performed. The median procedural time was 130 (110; 155) min with an LA time of 90 (75; 110) min. The fluoroscopy time was 22 (17; 27) min, and the median dose area product was 1829 (1044; 3099) cGycm^2^. Of all 473 patients undergoing CBA, 166 (35.1%) experienced AF recurrence during follow‐up. The median follow‐up time was 19 months.

### 3.2 LAA morphology and outcome

**FIGURE 1:**
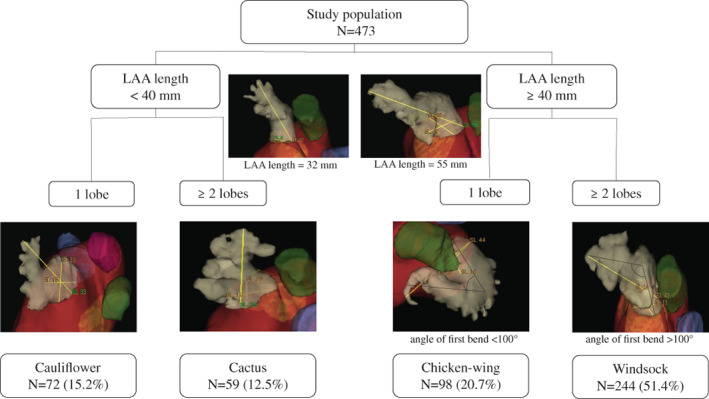
Classification of LAA morphology. This figure depicts the LAA morphological types as defined by Wang [22] with Kimura's quantitative qualifiers [23]. According to the measured LAA length and number of lobes, LAA morphology was classified into one of four types: windsock, chicken‐wing, cactus, and cauliflower. The prevalent LAA type distribution in the study is provided here. LAA, left atrial appendage

**FIGURE 2:**
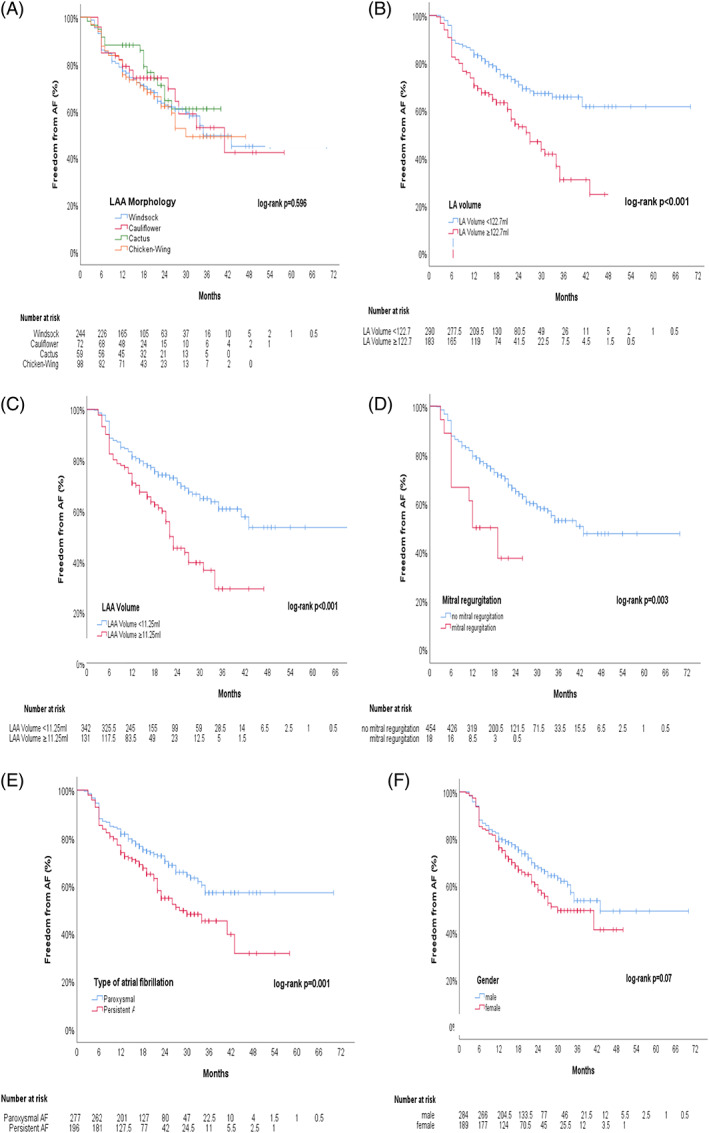
Outcome of cryoballoon ablation related to clinical predictors and LA/LAA anatomy. Kaplan–Meier curves demonstrate freedom of AF after cryoballoon ablation according to (A): LAA morphology, which showed no statistical impact on the recurrence rate of AF after cryoballoon ablation (log‐rank; *p* = 0.596). (B): LA volume with a cut‐off level of ≥122.7 ml: Larger LA volumes demonstrated a highly significant impact on the recurrence rate of AF after cryoballoon ablation (*p* < 0.001). (C): LAA volume cut‐off level of ≥11.25 ml: Larger LAA volumes demonstrated a highly significant impact on the recurrence rate of AF after cryoballoon ablation (*p* < 0.001). (D): mitral valve regurgitation ≥°II. Its presence was significantly related to a higher recurrence rate (log‐rank; *p* = 0.003). (E): AF‐type: Persistent type AF demonstrated a significant impact on the recurrence rate of AF after cryoballoon ablation (*p* = 0.001). (F): female sex on its own showed a non‐significant trend on the recurrence rate of AF after cryoballoon ablation (*p* = 0.07). AF, atrial fibrillation; LAA, left atrial appendage; LA, left atrium

The distribution of the LAA morphological types is shown in Figure 1. Among the 166 recurrence events, chicken‐wing morphology had the highest chance of recurrence at 37.8%, followed by windsock at 36.5%, cauliflower at 32.0%, and cactus at 28.8%. None of these categories was found to have a statistically significant impact on the AF recurrence rate (log‐rank; *p* = 0.596). The corresponding Kaplan–Meier plot is illustrated in Figure 2A.

### 3.3 LAA volume and the correlation to LA volume

LAA and LA volumes showed statistically significant correlations. We conducted linear regression to quantify the correlation of the LA volume with the LAA volume, which demonstrated that the LAA volume increased by 0.70 ml per 10 ml increase in LA volume (*p* < 0.001, see Figure S2).

### 3.4 LAA morphology

Chicken‐wing morphology had the largest LAA volume with 9.9 [7.98; 12.83] ml, followed by windsock morphology with an LAA volume of 9.65 [7.73; 13.08] ml. Cactus and cauliflower morphologies were smaller, with LAA volumes of 5.4 [4.6; 7.5] ml and 5.6 [4.4; 7.6] ml, respectively (*p* < 0.001).

### 3.5 Outcome predictors: Univariate analysis

In univariate Cox regression models, four continuous parameters were significant predictors of AF recurrence, with LA volume in ml demonstrating the highest predictive value (hazard ratio [HR] 1.01; 95% confidence interval [CI] [1.006–1.015]; *p* < 0.001). The LA volume determined by CCTA seemed to be superior compared to the LA diameter determined by transthoracic echocardiography and showed a higher significance (HR 1.037; 95% CI [1.015–1.060]; *p* = 0.001). The second most important parameter was the septum orifice distance (HR 1.053; 95% CI [1.028–1.08]; *p* < 0.001), followed by the trapezoid area of the posterior LA wall (HR 1.001; 95% CI [1–1.001]; *p* < 0.001) and the LAA volume (HR 1.051; 95% CI [1.025–1.078]; *p* < 0.001). All significant results are listed in Table 2 and Table S1.

**TABLE 2:** Univariate analysis of baseline characteristics and measurement data

	Univariate analysis		
	HR	95% CI	*p*‐value
Baseline characteristics			
Female sex	1.31	0.97–1.78	0.08
Age, years	1.02	1.000–1.034	0.05
Persistent AF	1.54	1.140–2.090	0.006
Mitral regurgitation ≥ II	2.5	1.31–4.75	0.005
Structural heart disease	1.38	1.01–1.88	0.04
LA diameter^a^, mm	1.037	1.015–1.060	0.002
LAA morphology			
Chicken‐wing	1.13	0.781–1.624	0.53
Windsock	1.09	0.802–1.476	0.59
Cactus	0.73	0.440–1.201	0.21
Cauliflower	0.94	0.604–1.459	0.78
Significant LAA measurements			
LAA max width, mm	1.03	1.003–1.049	0.03
LAA maximal depth, mm	1.02	1.000–1.036	0.05
LAA volume, ml	1.05	1.025–1.078	<0.0001
LAA Dmax 3D, mm	1.06	1.021–1.107	0.003
LAA Dmin 3D, mm	1.02	1.006–1.032	0.004
LAA Length, mm	1.02	1.002–1.041	0.03
Perimeter LAA ostium, mm	1.04	1.008–1.078	0.02
Area LAA ostium, mm^2^	1.20	1.100–1.300	0.001
Significant LA measurements			
LA volume, 10 ml	1.100	1.060–1.150	<0.000001
Roof top line, mm	1.03	1.013–1.050	0.001
Roof bottom line, mm	1.03	1.012–1.054	0.002
Posterior wall box height, mm	1.03	1.003–1.065	0.03
Distance MVA‐LA roof, mm	1.04	1.014–1.064	0.002
Depth of the LA, mm	1.04	1.013–1.067	0.003
Septum orifice distance, mm	1.05	1.028–1.080	<0.0001
Perimeter LIPV ostium, mm	1.02	1.001–1.042	0.04
Trapezoid area of the posterior LA wall, cm^2^	1.08	1.040–1.100	<0.0001

*Note*: This table illustrates the results of the univariate analysis of all important baseline characteristics and CCTA derived data.

Abbreviations: AF, atrial fibrillation; CCTA, cardiac computed tomography angiography; CI, confidence interval; Dmax, maximal ostial diameter; Dmin, minimal ostial diameter; HR, hazard ratio; LA, left atrium; LAA, left atrial appendage; LIPV, left inferior pulmonary vein; MVA, mitral valve annulus.

^a^ Measured by transthoracic echocardiography.

### 3.6 Outcome predictors: Correlation analysis

To avoid problems of multicollinearity in multivariate models, correlation analysis was performed for LA volume and all baseline parameters and measurement data according to the Pearson and Spearman tests (see Table S2, Table S3). Among the baseline parameters, mitral regurgitation, AF type, sex, age, structural heart disease, and hypertensive heart disease were significantly correlated to LA volume (the correlation matrix is provided in the Table S2). All CCTA results that related logically to the LA volume, such as the septum orifice distance, depth of the LA, distance of the mitral valve annulus to the LA roof, and trapezoid area of the posterior left atrial wall, showed significant positive correlations, as did the LAA volume and its companion parameters (e.g., perimeter of the LAA ostium or area of the LAA ostium). Details are provided in the Table S3. LA volume was the best CCTA‐derived measurement parameter for predicting AF recurrence and was, therefore, included in multivariate regression analysis.

### 3.7 Outcome predictors: Multivariate analysis

After precise analysis of intervariable correlation, a stepwise multivariate Cox‐regression model that included all significant baseline parameters and continuous CCTA parameters was performed. The independent parameters we evaluated were LA volume (HR 1.012; 95% CI [1.008–1.016]; *p* < 0.001, mitral regurgitation ≥°II (HR, 2.27; 95% CI [1.189 to 4.333]; *p* = 0.013), and female sex (HR 1.648; 95% CI, 1.196 to 2.271; *p* = 0.002). The exact values are presented in Table 3. All independent risk factors were evaluated using Kaplan–Meier Survival curves for AF recurrence during follow‐up (see Figure 2).

**TABLE 3:** Evaluation of independent risk factors^b^

	Univariate analysis			Multivariate analysis		
	HR	95% CI	*p*‐value	HR	95% CI	*p*‐value
Baseline characteristics						
Female sex	1.31	0.97–1.78	0.08	1.648	1.196–2.271	0.002
Age, years	1.02	1.000–1.034	0.05			
Persistent AF	1.54	1.140–2.090	0.006			
Mitral regurgitation ≥°II	2.5	1.31–4.75	0.005	2.270	1.189–4.33	0.013
Structural heart disease	1.38	1.01–1.88	0.04			
LA diameter^a^, mm	1.037	1.015–1.060	0.002			
CCTA measurements						
LA volume, 10 ml	1.100	1.060–1.150	<0.001	1.012	1.008–1.016	<0.001

Abbreviations: AF, atrial fibrillation; CCTA, cardiac computed tomography angiography; CI, confidence interval; HR, hazard ratio; LA, left atrium.

^a^ Determined by transthoracic echocardiography.

^b^ Univariate analysis of baseline characteristics and measurement data according freedom of AF after cryoballoon ablation provided multiple highly significant parameters. To prevent multicollinearity in the multivariate analysis, CCTA measurements were reduced to LA volume as it represents the most significant univariate parameter and showed highly significant correlation to all other measurement parameters. After stepwise multivariate regression with bidirectional elimination, three parameters could be identified as independent risk factors: female sex, mitral regurgitation ≥°II and LA volume. The statistical significance of the multivariate Cox regression model was *p* < 0.000001.

### 3.8 LA and LAA volume as dynamic predictors for AF recurrence

The cut‐off values for the LA and LAA volumes were determined using ROC analysis. LA volumes ≥122.7 ml (sensitivity 0.53, specificity 0.69, AUC 0.63) and LAA volumes ≥11.25 ml (sensitivity 0.39, and specificity 0.79, AUC 0.59) were associated with AF recurrence. Both cut‐off values showed high statistical significance (LA volume [log‐rank; *p* < 0.001] and LAA volume [log‐rank; *p* < 0.001]). The values are depicted in the Kaplan–Meier plots in terms of AF recurrence (Figure 2B,C).

### 3.9 Relationship of persistent atrial fibrillation and LAA volume

Persistent AF patients showed both larger LA volumes and LAA volumes as compared to patients with paroxysmal AF (108.2 [92.5; 126.4] ml versus 124.8 [103.3; 148.5], *p* < 0.001 and 8.3 [6.1; 13.9] ml versus 9.2 [7.2; 12.3] ml, *p* = 0.005).

### 3.10 Gender differences in LA and LAA volumes

The median LA volume was significantly smaller in females as compared to males (108.0 [92.4; 147.1] mL versus 118.1 [102.7; 131.0] ml, *p* < 0.001), while the median LAA volume of females was similar compared to males (8.4 [5.9; 11.1] ml versus 9.0 [6.8; 12.0] ml, *p* = 0.09).

## 4 DISCUSSION

To the best of our knowledge, this is the largest trial evaluating the impact of LAA anatomy and morphology, determined by preprocedural CCTA, on the recurrence of atrial arrhythmias after PVI.

LAA morphology was not associated with AF recurrence after cryoballoon PVI. This finding is in contrast to that of Kocyigit et al. who identified a relationship between cauliflower‐type LAA morphology and recurrences after CBA.^19^ In this sense, therefore, our study must be considered negative. Nevertheless, secondary and exploratory analyses strongly suggest that LAA size has a role in AF recurrence following PVI. First, LA and LAA volumes represent the two major metrics with respect to their ability to predict AF recurrence after PVI. Second, this is the first study to demonstrate a strong correlation between LA and LAA volumes. Third, owing to the large size of the study population, significant cut‐off values for LA and LAA volumes as predictors of recurrences were calculated. Fourth, female sex was an independent risk factor for AF recurrence, and gender differences for LA volumes were observed.

LA volume is the unique independent predictor of AF recurrence, whereas LAA volume is dependent on LA size. The risk of recurrence increases by 10% per 10 ml increase of the LA volume. Similarly, with each 1 ml increase of LAA volume, the risk of recurrence increases by 5.1%. Revealed cut‐off values regarding AF recurrence show that especially patients with LA volumes ≥122.7 ml or LAA volumes ≥11.25 ml are at risk for AF recurrence despite PVI. Interestingly, Teixeira et al. found the LAA volume>8.825 ml to be predictive for recurrence following radiofrequency catheter ablation of AF.^20^ Hence, the observation that LAA size plays a role in outcomes of AF ablation seems to be generalisable to ‘PVI only’ strategies in the initial procedure irrespectively of the used ablation technique.

Notably, size differences were observed between the LAA morphology types in the present study. The chicken‐wing and windsock types were larger than the cactus and cauliflower LAA types. However, even in the pooled analysis of the smaller LAA morphology types (cactus and cauliflower) compared with the larger LAA morphology types (chicken‐wing and windsock), there was no association of LAA shape and recurrences. Neither PV size nor PV variations such as a common ostium or additional right PV were associated with recurrence, which confirms the findings of Khoueiry et al.^21^

LA volume measured by CCTA had a superior predictive value as compared to LA diameter determined by echocardiography. According to the literature, there is no outcome benefit if a CCTA is performed prior to the procedure^22^ and the indication for PVI is based on clinical findings.^1^ Other important advantages considering echocardiography over pre‐procedural CCTA include the absence of radiation, the low costs and the low effort. However, CCTA before the AF ablation procedure may be beneficial for navigation and the identification of PV variants or for the exclusion of CAD as a cause of AF.^1^ CCTA might also be used to rule out thrombi prior to ablation as a potential alternative to transesophageal echocardiography.^1^, ^14^ It is unknown if LA volume measured by TTE is equally predictive for recurrence after CBA as compared to CCTA LA volume. However, in a multivariate Cox‐regression model not including CCTA measurement data, independent clinical predictors of recurrence were mitral insufficiency ≥II°, female sex, AF type, and the LA diameter measured by echocardiography. Although this is a weaker model compared to the Cox‐regression model using CCTA measurements, it also yields the most important information for the risk assessment of AF recurrence, enables determining the potential benefit of ablation, and should be used in clinical practice.

Female sex has already been described as a clinical predictor of recurrence,^23^ but meta‐analyses have shown ambiguous evidence for sex‐related differences in outcomes with AF ablation.^1^ Our results indicated that female sex was an independent risk factor for AF recurrence after CBA. Female patients were older and underrepresented compared with male patients. These results are consistent with those of all AF ablation trials. However, age was not an independent predictor for AF recurrence after CBA, which is in line with the recently published data.^24^, ^25^ Interestingly, LA volume was significantly smaller in females, while the LAA volume in females was similar to that in males. Hypothetically, sex‐related differences in the presence of fibrotic atrial myopathy or additional extra‐PV trigger sites may explain our observations. Ultimately, the answer to these questions may lead to specific, sex‐tailored AF ablation strategies.

The outcome of a ‘PVI only’ strategy in the first ablation procedure is sufficient for the majority of patients, as confirmed by our results. Based on the present study, patients with a large LA volume are more likely to also have large LAA volumes. The LAA is a potential extra‐PV trigger. Hypothetically, a larger LAA might yield AF triggers more frequently. During AF ablation, however, the identification of extra‐PV triggers is challenging. Strategies for repeat ablation, if all PVs are isolated, must be evaluated further, and studies evaluating the effect of LAA electrical isolation as well as effects on major outcome parameters are underway. However, this concept does expose the patients to additional thromboembolic risk,^26^ and extensive LA ablation is associated with a higher rate of acute complications, such as cardiac tamponade.^27^ In the interventional treatment of AF, the preprocedural identification of patients who would benefit from additional ablation beyond PVI would be of significance. Preprocedural CCTA might be a useful imaging modality if LAA dimensions are of interest. For example, this may be important in studies evaluating ablation strategies for patients with recurrences despite successful PVI.

## 5 STUDY LIMITATIONS

This was a single‐centre observational study with inherent limitations accompanying this type of study design. The data are not fully consecutive because not every patient underwent cardiac CT before the AF ablation procedure, and image quality was not sufficient for LAA assessment in every patient. In addition, LA and LAA volume measurement is influenced by the hydration status of the patient.^28^ Although the CCTA was carried out on sober patients, we cannot exclude the possibility of dehydration and different volume loadings among the patients.

Results were based on clinical visits and the evaluation of symptoms, ECG, and Holter monitoring (1–7 days) during routine follow‐up. No systematic, continuous monitoring with implantable devices was available. Therefore, asymptomatic recurrences might have been missed. Furthermore, information as to whether the LAA was arrhythmogenic during the CBA procedure was not available; therefore, we were unable to correlate LAA size or morphology with the incidence of LAA triggers.

## 6 CONCLUSION

LAA volume was strongly correlated to LA volume and was a dependent predictor of recurrence after cryoballoon PVI. The main independent predictors were LA volume, female sex, and mitral regurgitation ≥°II. LA volume was significantly smaller in females, while the LAA volume in females was similar to that in males. LAA shape was no predictor of arrhythmia free survival. The role of LAA in AF treatment should be evaluated in further studies. Preprocedural CCTA might be a useful imaging modality if LAA dimensions are of interest, in studies evaluating ablation strategies for patients with recurrences despite successful PVI.

## CONFLICT OF INTEREST

Dr. Straube received honoraria for lectures from Medtronic, Astra Zeneca, Bristol‐Myers‐Squibb, I.Med.Pro, outside the submitted work; educational support from Pfizer. Dr. Dorwarth reports honoraria for lectures from Medtronic Inc., outside the submitted work. Dr. Hoffmann is head of the department; the department received compensation for participation in clinical research trials outside the submitted work from Abbott, Bayer, Biotronik, Boehringer Ingelheim, Edwards, Elixier, Medtronic, and Stentys. Dr. Hartl participates in the EP fellowship from Boston Scientific, received educational support from Biotronik, Daiichi Sankyo and honoraria for lectures from Bristol Myers Squibb outside the submitted work. Dres. Brück, Crispin, Ebersberger, Helmberger, Pongratz, Tesche, and Wankerl have nothing to declare.

## Supporting information


Figure S1 Study population – selection criteria
This flow chart explains the selection process of the study population. The top box shows the number of all patients included at the beginning. Each branching demonstrates one step of selection.
CCTA: cardiac computed tomography angiography

Figure S2 Correlation of LA and LAA volumes
The figure shows a linear regression model of LAA volume and LA volume. It demonstrates that per 10 mL increase of LA volume, LAA volume increases by 0.6 mL. The significance level of the model was p < 0.001.
LAA: left atrial appendage; LA: left atrium

Figure S3 Schematic demonstration of important LA and LAA measurements
The picture in the back represents a three‐dimensional left atrium in anterior view with important anatomical components. To improve the illustration of the measurements, a two‐dimensional overlying scheme was added. The dotted lines and their respective numbers indicate the different measurements.
LAA: left atrial appendage; LA: left atrium; LSPV: left superior pulmonary vein; LIPV: left inferior pulmonary vein; RSPV: right superior pulmonary vein; RIPV: right inferior pulmonary vein; 1: LAA length; 2: distance to the first bend; 3: septum‐orifice distance; 4: distance of the mitral valve annulus to the LA roof; 5: roof bottom line; 6: roof top line; 7: posterior wall box height

Appendix S1: Supporting information

## Data Availability

The data that support the findings of this study are available from the corresponding author upon reasonable request.
