# Supplementary figures and images for: Cardiac computed tomography angiography‐derived analysis of left atrial appendage morphology and left atrial dimensions for the prediction of atrial fibrillation recurrence after pulmonary vein isolation
Source: Clin Cardiol. 2021 Oct 14;44(11):1636–45. doi: 10.1002/clc.23743 (PMC8571558; doi:10.1002/clc.23743)

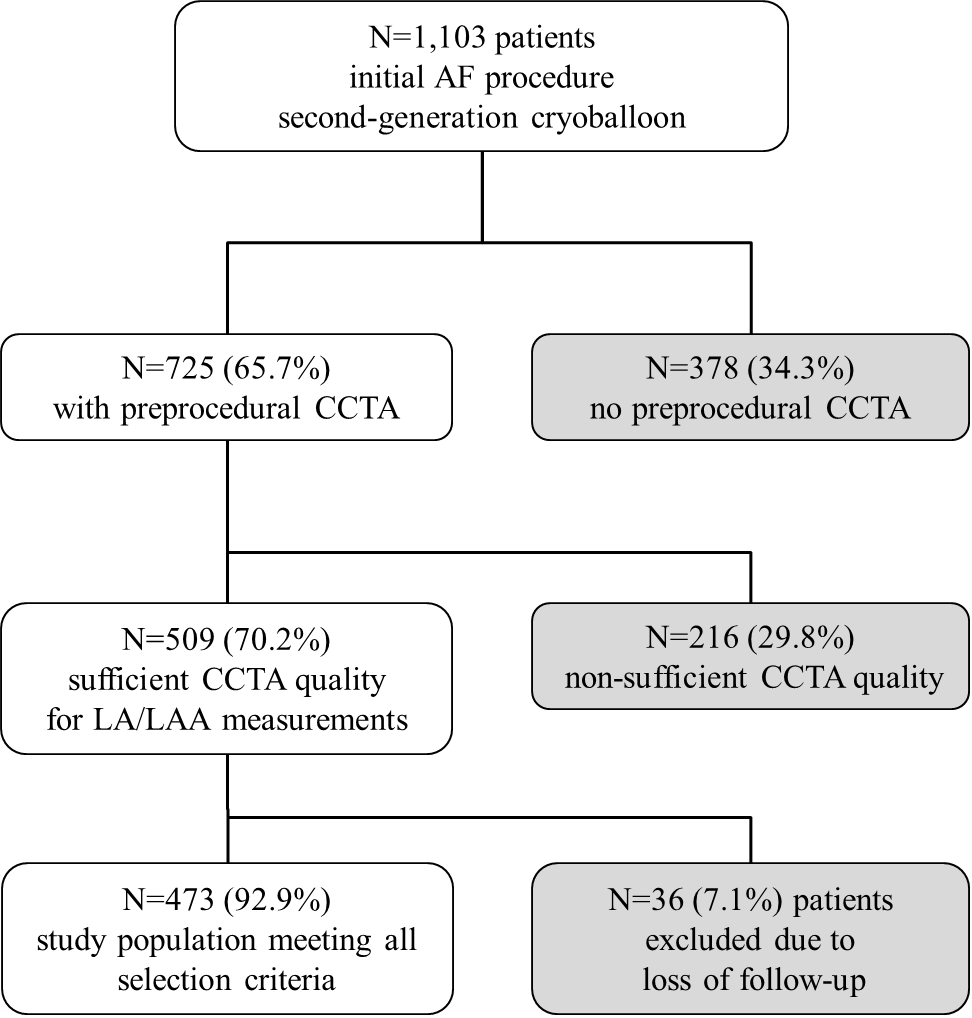

Supplement: Supplementary file 1 — Figure S1 Study population – selection criteria This flow chart explains the selection process of the study population. The top box shows the number of all patients included at the beginning. Each branching demonstrates one step of selection. CCTA: cardiac computed tomography angiography [file CLC-44-1636-s003.docx]

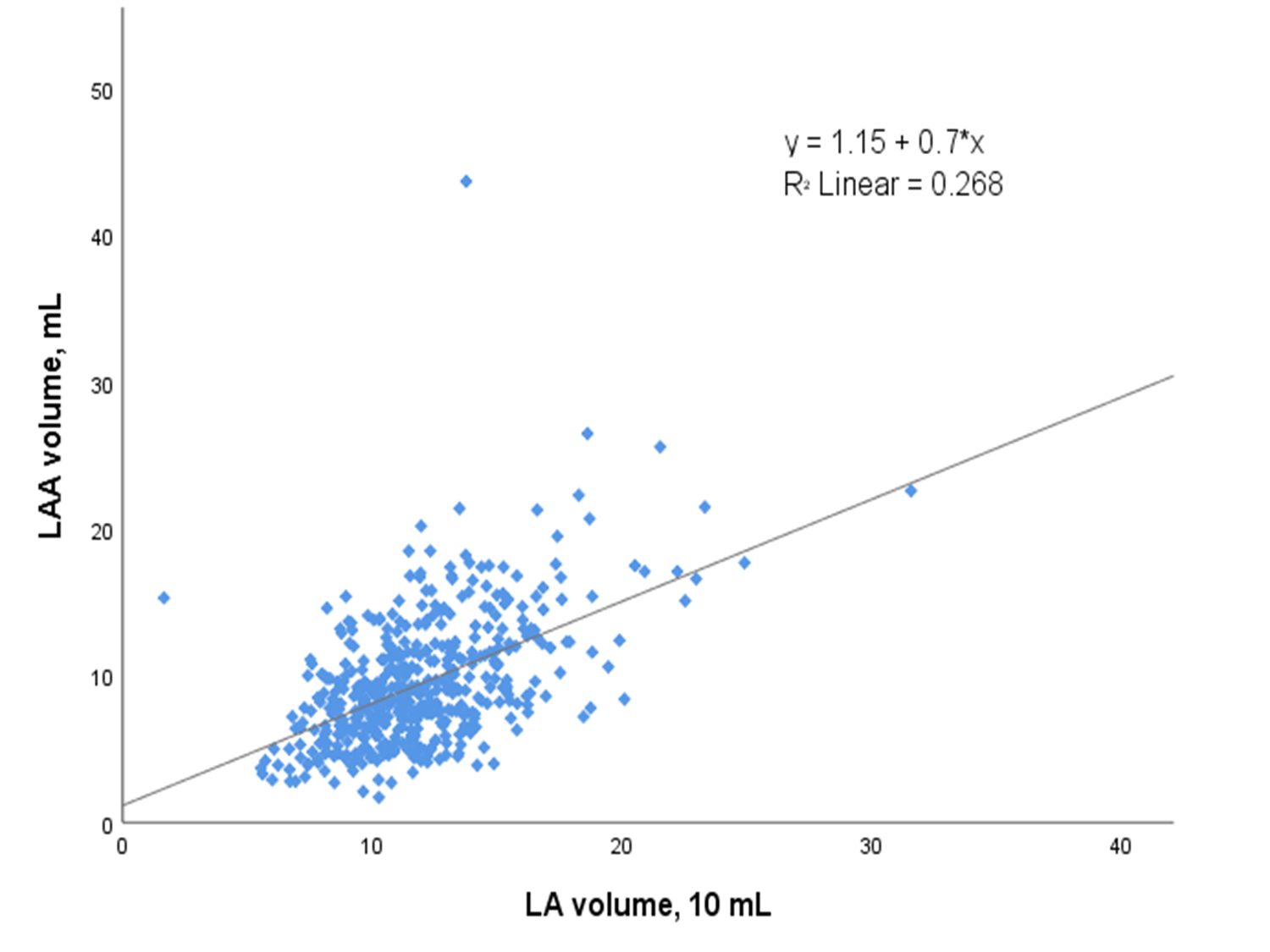

Supplement: Supplementary file 2 — Figure S2 Correlation of LA and LAA volumes The figure shows a linear regression model of LAA volume and LA volume. It demonstrates that per 10 mL increase of LA volume, LAA volume increases by 0.6 mL. The significance level of the model was p < 0.001. LAA: left atrial appendage; LA: left atrium [file CLC-44-1636-s002.docx]

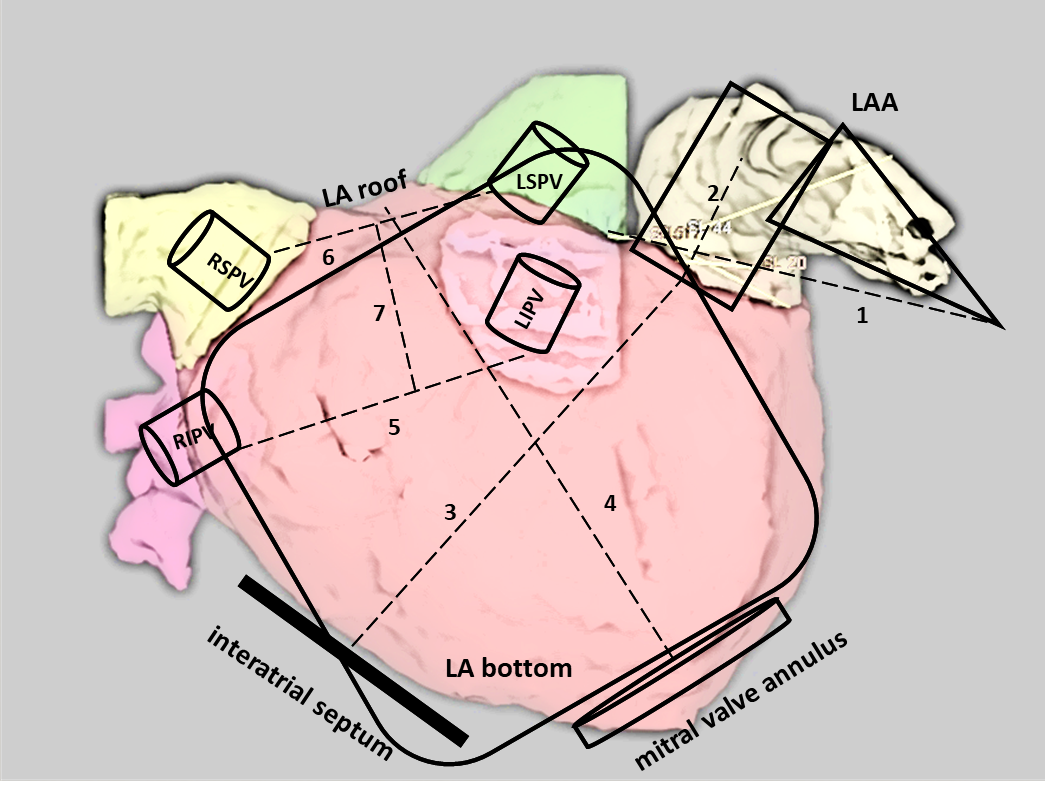

Supplement: Supplementary file 3 — Figure S3 Schematic demonstration of important LA and LAA measurements The picture in the back represents a three‐dimensional left atrium in anterior view with important anatomical components. To improve the illustration of the measurements, a two‐dimensional overlying scheme was added. The dotted lines and their respective numbers indicate the different measurements. LAA: left atrial appendage; LA: left atrium; LSPV: left superior pulmonary vein; LIPV: left inferior pulmonary vein; RSPV: right superior pulmonary vein; RIPV: right inferior pulmonary vein; 1: LAA length; 2: distance to the first bend; 3: septum‐orifice distance; 4: distance of the mitral valve annulus to the LA roof; 5: roof bottom line; 6: roof top line; 7: posterior wall box height [file CLC-44-1636-s004.docx]
