# Supplementary material for: Cardiac computed tomography angiography‐derived analysis of left atrial appendage morphology and left atrial dimensions for the prediction of atrial fibrillation recurrence after pulmonary vein isolation
Source: Clin Cardiol. 2021 Oct 14;44(11):1636–45. doi: 10.1002/clc.23743 (PMC8571558; doi:10.1002/clc.23743)
Supplement: Supplementary file 4 — Appendix S1: Supporting information [file CLC-44-1636-s001.docx]

**Supplement Appendix**

**Cryoballoon Ablation Procedure**

PVI was performed using the 28-mm CB as the preferred balloon size (CBG2: Arctic Front Advance, Medtronic Inc., Minneapolis, MN, USA), which was introduced into the LA over a 15F steerable sheath (FlexCath Advance™, Medtronic Inc., Minneapolis, MN, USA). If PVI was not achieved, the 23-mm CB size was allowed exclusively for small PV ≤21 mm. Radiofrequency (RF) or cryo-tip touch-up applications were allowed if necessary, to achieve PVI. During the procedure, a 3D reconstruction of the LA via CT was available to guide the operator and to identify PV variants. Transseptal puncture was guided by intracardiac echocardiography (ICE; Vivid I, GE Healthcare EUROPE, GE Ultraschall Deutschland GmbH, Solingen, Germany). ICE was also used to assure optimal balloon positioning, vessel occlusion, and to visualize diaphragmatic motion during ablation of the right-sided PVs if palpation of the diaphragm was difficult. An 8-pole micro-circular mapping catheter (20-mm Achieve™ or Achieve Advance™ Mapping Catheter, Medtronic Inc.) was used for CB positioning and the assessment of real-time PV potentials. After detection of adequate vessel occlusion by PV angiography, the refrigerant supply was started and PVI was performed with at least one freeze-thaw-freeze cycle per vein. If PV potentials could be identified during the ablation procedure, the time to PV isolation (TTI) was determined. An additional application was applied in those veins with TTI ≥45 s or if no TTI was detected. The standard application time was between 180 and 240 s. For safety measures, the temperature limit for right sided PV was -55 °C. In addition, PV potentials were mapped before and after each freeze cycle and again 15 min at the end of the last freeze. An endoluminal esophageal temperature probe (SensiTherm™, St. Jude Medical, Saint Paul, MN, USA) was used in all patients with a cut-off of ≤+15 °C. Phrenic nerve (PN) pacing (1200 ms cycle length) and manual diaphragm examination was performed to reduce the risk of PN palsy during ablation of the right PVs. Intravenous heparin administration ensured an activated clotting time between 300 and 400 s during the entire procedure with measurements taken at least every 30 min while in the LA. Prior to sheath removal, protamine was given to reduce the bleeding risk. One hour after sheath removal, either continuous unfractionated heparin was started with a target partial thromboplastin time of 50-70 s or novel oral anticoagulants were restarted immediately. Finally, oral anticoagulation was restarted on the day after the procedure and continued for at least 3 months according to the CHA_2_DS_2_VaSc score.

**Tables**

**Supplementary Table 1: Detailed CCTA results of LA and LAA anatomy**

|  | **All patients** | | **With recurrence** | | **Without recurrence** | | **p-value** |
| --- | --- | --- | --- | --- | --- | --- | --- |
|  | Value | Analyzed | Value | Analyzed | Value | Analyzed |  |
|  |  |  |  |  |  |  |  |
| **LAA morphology** | | | | | | | |
| **Chicken-wing** | 98 (20.7) | 473 | 37 (7.8) | 166 | 61 (12.9) | 307 | 0.53 |
| **Windsock** | 244 (51.6) | 473 | 89 (18.8) | 166 | 155 (32.8) | 307 | 0.59 |
| **Cactus** | 59 (12.5) | 473 | 17 (3.6) | 166 | 42 (8.9) | 307 | 0.21 |
| **Cauliflower** | 72 (15.2) | 473 | 23 (4.9) | 166 | 49 (10.4) | 307 | 0.78 |
|  |  |  |  |  |  |  |  |
| **LAA measurements** | | | | | | | |
| **LAA max width, mm** | 37.36±6.71 | 473 | 38.16±6.79 | 166 | 36.93±6.6 | 307 | 0.029 |
| **LAA maximal depth, mm** | 39.98±8.73 | 473 | 41.08±9.32 | 166 | 39.38±8.35 | 307 | 0.047 |
| **LAA maximal height, mm** | 26.8 [22.84; 30.90] | 473 | 27.38 [23; 31,84] | 166 | 26.44 [22.67; 30.5] | 307 | 0.21 |
| **LAA Volume, mL** | 8.7 [6.5; 11,6] | 473 | 9.4 [7.1; 13.4] | 166 | 8.3 [6.3; 11] | 307 | <0.001 |
| **LAA Dmax 3D, mm** | 25 [22; 28] | 473 | 25 [21.8; 29] | 166 | 25 [22; 27] | 307 | 0.015 |
| **LAA Dmin 3D, mm** | 18 [15; 20] | 473 | 18 [16; 21] | 166 | 16 [15; 20] | 307 | 0.003 |
| **LAA Length, mm** | 44.4±7.8 | 473 | 45.3±8.2 | 166 | 43.8±7.6 | 307 | 0.03 |
| **Perimeter LAA ostium, mm** | 67.92±12.18 | 473 | 69.96±13.35 | 166 | 66.81±11.37 | 307 | 0.004 |
| **Area LAA ostium, mm²** | 212.01 [128.4; 263.9] | 473 | 362.9 [280.4; 462] | 166 | 326.7 [263.9; 417.8] | 307 | 0.001 |
|  |  |  |  |  |  |  |  |
| **LA Measurements** | | | | | | | |
| **LA Volume, mL** | 115.1 [97.2; 135.2] | 473 | 124 [103.9; 147.1] | 166 | 110.2 [95.2; 129.5] | 307 | <0.001 |
| **Roof Top line, mm** | 35 [31; 41] | 473 | 37.5 [31; 43] | 166 | 34 [30; 39] | 307 | 0.001 |
| **Roof Bottom line, mm** | 42 [37; 46] | 473 | 43 [38.8; 47.3] | 166 | 41 [36; 45] | 307 | 0.002 |
| **Posterior wall box height, mm** | 32 [29; 35] | 473 | 32 [29; 36] | 166 | 32 [29; 34] | 307 | 0.031 |
| **Distance MVA-LA roof, mm** | 69 [65; 74] | 473 | 70 [65.8; 75] | 166 | 68 [65; 73] | 307 | 0.002 |
| **Depth of the LA, mm** | 38.53±5.9 | 473 | 39.61±6.3 | 166 | 37.95±5.6 | 307 | 0.003 |
| **Septum orifice distance, mm** | 58 [54; 61] | 473 | 59 [55; 63] | 166 | 57 [53; 60] | 307 | <0.001 |
| **Trapezoid area of the posterior LA wall, cm²** | 12.05 [10.32; 14.23] | 473 | 12.87 [10.76; 17.28] | 166 | 11.78 [10.08; 15.79] | 307 | <0.001 |
| **Width of the LAA ridge, mm** | 4 [3, 5] | 473 | 4 [3; 6] | 166 | 4 [3; 5] | 307 | 0.60 |
|  |  |  |  |  |  |  |  |
| **PV** |  |  |  |  |  |  |  |
| **LSPV maximal ostial diameter, mm** | 21 [19; 23] | 473 | 21 [19; 23] | 166 | 21 [19; 23] | 307 | 0.87 |
| **LSPV minimal ostial diameter, mm** | 14 [12; 16] | 473 | 15 [12.8; 17] | 166 | 14 [12; 16] | 307 | 0.17 |
| **LSPV ostial perimeter, mm** | 56 [52; 61.6] | 473 | 55.8 [51.9; 62.6] | 166 | 56.2 [51.9; 61.6] | 307 | 0.55 |
| **LSPV ostial area, cm^2^** | 2.3 [1.9; 2.9] | 473 | 2.3 [1.9; 3] | 166 | 2.3 [2.8] | 307 | 0.95 |
| **LIPV maximal ostial diameter, mm** | 17 [16; 19] | 473 | 18 [16; 20] | 166 | 17 [15.8; 19] | 307 | 0.028 |
| **LIPV minimal ostial diameter, mm** | 13 [11; 15] | 473 | 14 [11; 16] | 166 | 13 [11; 15] | 307 | 0.053 |
| **LIPV ostial perimeter, mm** | 48.5 [43.4; 53.6] | 473 | 50.2 [44; 55.2] | 166 | 47.4 [43.4; 53.2] | 307 | 0.044 |
| **LIPV ostial area, cm^2^** | 1.8 [1.4; 2.3] | 473 | 1.9 [1.3; 2.3] | 166 | 1.7 [1.4; 2.1] | 307 | 0.43 |
| **RIPV maximal ostial diameter, mm** | 18 (16; 20) | 473 | 18 [16; 20] | 166 | 18 [16; 21] | 307 | 0.54 |
| **RIPV minimal ostial diameter, mm** | 15 (13; 17) | 473 | 15 [13; 18] | 166 | 15 [13; 17] | 307 | 0.53 |
| **RIPV ostial perimeter, mm** | 52.3 (47.1; 59) | 473 | 53.4 [45.7; 59.9] | 166 | 52 [47.1; 58.1] | 307 | 0.64 |
| **RIPV ostial area, cm^2^** | 2.1 (1.7; 2.7) | 473 | 2.2 [1.6; 2.8] | 166 | 2.1 [1.7; 2.6] | 307 | 0.73 |
| **RSPV maximal ostial diameter, mm** | 21 (19; 24) | 473 | 21 [19; 23] | 166 | 21 [19; 24] | 307 | 0.93 |
| **RSPV minimal ostial diameter, mm** | 17 (14; 19) | 473 | 17 [14.3; 19] | 166 | 16 [14; 18] | 307 | 0.21 |
| **RSPV ostial perimeter, mm** | 60.2 (53.8; 66.5) | 473 | 60 [53.7; 66] | 166 | 59.8 [53.3; 66] | 307 | 0.61 |
| **RSPV ostial area, cm^2^** | 2.8 (2.2; 3.4) | 473 | 2.8 [2.2; 3.4] | 166 | 2.7 [2.1; 3.3] | 307 | 0.57 |
| **LCO maximal ostial diameter, mm** | 30.5±4.3 | 473 | 31 [27; 35] | 166 | 30 [27; 33] | 307 | 0.48 |
| **LCO minimal ostial diameter, mm** | 17.7±4.2 | 473 | 17 [14; 22.8] | 166 | 17 [15; 20] | 307 | 0.48 |
| **LCO ostial perimeter, mm** | 75.9 (69.1; 88) | 473 | 78.3 [68.3; 90.3] | 166 | 75 [69.3; 84.8] | 307 | 0.39 |
| **LCO ostial area, cm^2^** | 3.9 (3.3; 5.4) | 473 | 4.2 [3; 5.9] | 166 | 3.8 [3.3; 5.1] | 307 | 0.53 |
| **RMPV maximal ostial diameter, mm** | 9.5 (6; 12.3) | 473 | 9 [7.5; 10] | 166 | 12 [9; 13] | 307 | 0.64 |
| **RMPV minimal ostial diameter, mm** | 5.5 (4; 7.8) | 473 | 4 [4; 5] | 166 | 7 [6; 9] | 307 | 0.33 |
| **RMPV ostial perimeter, mm** | 30 (23.3; 41) | 473 | 28 [22.9; 30] | 166 | 39.5 [32.4; 42.7] | 307 | 0.047 |
| **RMPV ostial area, cm^2^** | 0.70 (0.43; 1.24) | 473 | 0.6 [0.4; 0.7] | 166 | 1.2 [0.9; 1.3] | 307 | 0.062 |

n (%), Mean ± SD, or Median (IQR)

LAA: left atrial appendage, LA: left atrium, MVA: mitral valve annulus, LSPV: left superior pulmonary vein, LIPV: left inferior pulmonary vein, RIPV: right inferior pulmonary vein, RSPV: right superior pulmonary vein, LCO: left common ostium, RMPV: right middle pulmonary vein, Dmax: maximal diameter, Dmin: minimal diameter.

**Supplementary Table 2: Correlation matrix of baseline characteristics and LA volume**

| **_Spearman_ ^Pearson^** | **1** | **2** | **3** | **4** | **5** | **6** | **7** | **8** | **9** | **10** | **11** | **12** | **13** | **14** | **15** | **16** | **17** | **18** | **19** | **20** | **21** |
| --- | --- | --- | --- | --- | --- | --- | --- | --- | --- | --- | --- | --- | --- | --- | --- | --- | --- | --- | --- | --- | --- |
| **1-LAAV, mL** |  | **,543 **** | **ns** | **ns** | **.146 *** | **.258 **** | **-.145 *** | **ns** | **ns** | **ns** | **ns** | **ns** | **.225 **** | **.145 *** | **ns** | **ns** | **ns** | **ns** | **ns** | **.168 *** | **ns** |
| **2-LAV, mL** | **.494 **** |  | **.159 *** | **-.171 *** | **ns** | **.526 **** | **-.276 **** | **ns** | **-.148 *** | **.207 **** | **ns** | **.167 *** | **ns** | **ns** | **ns** | **ns** | **ns** | **ns** | **ns** | **ns** | **ns** |
| **3-Age, years** | **ns** | **.197 **** |  | **.316 **** | **ns** | **.246 **** | **ns** | **ns** | **-.170 *** | **ns** | **ns** | **.158 *** | **.213 **** | **ns** | **ns** | **ns** | **ns** | **ns** | **ns** | **ns** | **ns** |
| **4-Females** | **ns** | **.164 *** | **.322 **** |  | **ns** | **ns** | **ns** | **.146 *** | **-.191 **** | **-.330 **** | **ns** | **ns** | **ns** | **ns** | **ns** | **-.161 *** | **ns** | **ns** | **ns** | **ns** | **ns** |
| **5-MG ≥°II** | **.238 **** | **ns** | **ns** | **ns** |  | **.146 *** | **ns** | **ns** | **ns** | **ns** | **ns** | **ns** | **.314 **** | **ns** | **.181 *** | **ns** | **ns** | **ns** | **ns** | **ns** | **ns** |
| **6-LADia, mm*** | **.237 **** | **.530 **** | **.267 **** | **ns** | **ns** |  | **-.181 *** | **ns** | **ns** | **.181 *** | **ns** | **.188 *** | **.275 **** | **.197 **** | **ns** | **.185 *** | **ns** | **ns** | **ns** | **ns** | **ns** |
| **7-EF (%)** | **ns** | **-.211 **** | **ns** | **ns** | **ns** | **-.210 **** |  | **ns** | **.229 **** | **-.392 **** | **ns** | **ns** | **-.242 **** | **ns** | **-.529 **** | **ns** | **ns** | **ns** | **ns** | **ns** | **ns** |
| **8-DM** | **ns** | **ns** | **ns** | **.146 *** | **ns** | **ns** | **ns** |  | **ns** | **ns** | **ns** | **ns** | **ns** | **ns** | **ns** | **ns** | **ns** | **ns** | **ns** | **ns** | **ns** |
| **9-GFR, mL/min** | **ns** | **-.173 *** | **ns** | **-.152 *** | **ns** | **ns** | **.228 **** | **ns** |  | **-.516 **** | **ns** | **-.219 **** | **-.155 *** | **ns** | **-.145 *** | **ns** | **ns** | **ns** | **ns** | **ns** | **ns** |
| **10-Crea, mg/dl** | **ns** | **.262 **** | **-.162 *** | **-.432 **** | **ns** | **.159 **** | **-.287 **** | **ns** | **-.387 **** |  | **ns** | **.154 *** | **.167 *** | **.176 *** | **.170 *** | **ns** | **ns** | **ns** | **ns** | **ns** | **ns** |
| **11-OSAS** | **ns** | **ns** | **ns** | **ns** | **ns** | **ns** | **ns** | **ns** | **ns** | **ns** |  | **.156 *** | **ns** | **ns** | **ns** | **ns** | **ns** | **.318 **** | **ns** | **ns** | **ns** |
| **12-aHT** | **ns** | **.175 *** | **.153 *** | **ns** | **ns** | **.202 **** | **ns** | **ns** | **-.186 **** | **ns** | **.155 *** |  | **.364 **** | **.373 **** | **ns** | **.209 **** | **ns** | **ns** | **ns** | **ns** | **ns** |
| **13-SHD** | **.220 **** | **ns** | **.198 **** | **ns** | **.314 **** | **.312 **** | **-.196 **** | **ns** | **ns** | **.142 *** | **ns** | **.364 **** |  | **.699 **** | **.234 **** | **ns** | **ns** | **ns** | **ns** | **ns** | **ns** |
| **14-HHD** | **ns** | **ns** | **ns** | **ns** | **ns** | **.223 **** | **-.185 **** | **ns** | **ns** | **.171 *** | **ns** | **.373 **** | **.699 **** |  | **ns** | **.186 *** | **ns** | **ns** | **ns** | **ns** | **ns** |
| **15-CM** | **ns** | **ns** | **ns** | **ns** | **.140 *** | **ns** | **-.260 **** | **ns** | **-.180** | **ns** | **ns** | **ns** | **.259 **** | **ns** |  | **ns** | **ns** | **ns** | **ns** | **ns** | **ns** |
| **16-OW (BMI>25)** | **ns** | **ns** | **ns** | **ns** | **ns** | **.189 *** | **ns** | **ns** | **ns** | **ns** | **ns** | **.209 **** | **ns** | **.186 *** | **ns** |  | **.374 **** | **.179 *** | **ns** | **ns** | **-.244 **** |
| **17-Obese °I (BMI>30)** | **ns** | **ns** | **ns** | **ns** | **ns** | **ns** | **ns** | **ns** | **ns** | **ns** | **ns** | **ns** | **ns** | **ns** | **ns** | **.374 **** |  | **.479 **** | **ns** | **ns** | **ns** |
| **18-Obese °II (BMI>35)** | **ns** | **ns** | **ns** | **ns** | **ns** | **ns** | **ns** | **ns** | **ns** | **ns** | **.318 **** | **ns** | **ns** | **ns** | **ns** | **.179 *** | **.479 **** |  | **ns** | **ns** | **ns** |
| **19-LCO^#^** | **ns** | **ns** | **ns** | **ns** | **ns** | **ns** | **.159 *** | **ns** | **ns** | **ns** | **ns** | **ns** | **ns** | **ns** | **ns** | **ns** | **ns** | **ns** |  | **ns** | **ns** |
| **20-AV^#^** | **.149 *** | **ns** | **ns** | **ns** | **ns** | **ns** | **ns** | **ns** | **ns** | **ns** | **ns** | **ns** | **ns** | **ns** | **ns** | **ns** | **ns** | **ns** | **ns** |  | **ns** |
| **21-AFH, months** | **.182 *** | **ns** | **ns** | **ns** | **ns** | **ns** | **ns** | **ns** | **ns** | **ns** | **ns** | **ns** | **ns** | **ns** | **ns** | **-.204 *** | **ns** | **ns** | **ns** | **ns** |  |

This table depicts all significant correlation coefficients regarding the baseline characteristics and LAA volume. The values on the top represent the calculated correlation coefficients by Spearman, the values on the bottom by Pearson, respectively.

LAAV: left atrial appendage volume; LAV: left atrial volume; HHD: hypertensive heart disease; LADia: left atrial diameter by echocardiography; EF: ejection fraction; DM: diabetes mellitus; GFR: glomerular filtration rate; Crea: Creatinine; OSAS: obstructive sleep apnea; SHD: structural heart disease; MG > °II: Mitral regurgitation > °II; aHT: arterial hypertension; CM: Cardiomyopathy; LCO: left common ostium; AV: accessory pulmonary veins; AFH: atrial fibrillation history

*p<0.05; **p<0.01

**Supplementary table 3: Correlation matrix of CCTA measurement data**

| **_Spearman_ ^Pearson^** | **1** | **2** | **3** | **4** | **5** | **6** | **7** | **8** | **9** | **10** | **11** | **12** | **13** | **14** | **15** | **16** |
| --- | --- | --- | --- | --- | --- | --- | --- | --- | --- | --- | --- | --- | --- | --- | --- | --- |
| **1-LAAV, mL** |  | **,543 **** | **ns** | **.303 **** | **-.261 **** | **-.348 **** | **.710 **** | **.698 **** | **.782 **** | **.666 **** | **.758 **** | **.812 **** | **.807 **** | **.424 **** | **.351 **** | **.261 **** |
| **2-LAV, mL** | **.494  **** |  | **ns** | **ns** | **-.194 **** | **ns** | **.294 **** | **.353 **** | **.443 **** | **.492 **** | **.406 **** | **.511 **** | **.527 **** | **.724 **** | **.704 **** | **.689 **** |
| **3-Chicken-Wing** | **.178 *** | **.150 *** |  | **-.558 **** | **-.179 *** | **-.225 **** | **.183 *** | **ns** | **.185 **** | **ns** | **ns** | **.188 **** | **.172 *** | **.191 **** | **ns** | **.167 *** |
| **4-Windsock** | **.312 **** | **ns** | **-.558 **** |  | **-.355 **** | **-.447 **** | **.265 **** | **.521 **** | **.160 *** | **.149 *** | **.507 **** | **.173 *** | **.161 *** | **ns** | **.154 *** | **ns** |
| **5-Cactus** | **-.306 **** | **-.211 **** | **-.179 *** | **-.355 **** |  | **-.143 *** | **-.213 **** | **-.232 **** | **-.220 **** | **-.218 **** | **-.314 **** | **-.243 **** | **-.233 **** | **-.210 *** | **-.205 **** | **-.141 *** |
| **6-Cauliflower** | **-.381 **** | **ns** | **-.225 **** | **-.447 **** | **-.143 *** |  | **-.399 **** | **-.503 **** | **-.250 **** | **-.167 *** | **-.588 **** | **-.252 **** | **-.225 **** | **ns** | **ns** | **ns** |
| **7-LAA max width, mm** | **.746 **** | **.293 **** | **.190 **** | **.260 **** | **-.218 **** | **-.394 **** |  | **.458 **** | **.562 **** | **.427 **** | **.675 **** | **.565 **** | **.532 **** | **.241 **** | **.160 *** | **.166 *** |
| **8-LAA max depth, mm** | **.682 **** | **.323 **** | **ns** | **.535 **** | **-.283 **** | **-.509 **** | **.441 **** |  | **.488 **** | **.523 **** | **.791 **** | **.553 **** | **.548 **** | **.286 **** | **.273 **** | **ns** |
| **9-LAA Dmax ostium, mm** | **.754 **** | **.447 **** | **.197 *** | **.159 *** | **-.239 **** | **-.246 **** | **.553 **** | **.457 **** |  | **.661 **** | **.519 **** | **.945 **** | **.885 **** | **.344 **** | **.312 **** | **.204 **** |
| **10-LAA Dmin ostium, mm** | **.624 **** | **.509 **** | **.166 *** | **ns** | **-.239 **** | **-.183 *** | **.392 **** | **.493 **** | **.581 **** |  | **.484 **** | **.859 **** | **.910 **** | **.468 **** | **.330 **** | **.229 **** |
| **11-LAA Length, mm** | **.770 **** | **.380 **** | **.153 *** | **.521 **** | **-.376 **** | **-.582 **** | **.645 **** | **.792 **** | **.507 **** | **.482 **** |  | **.565 **** | **.546 **** | **.335 **** | **.297 **** | **.156 *** |
| **12-Perimeter LAA ostium, mm** | **.784 **** | **.527 **** | **.218 **** | **.160 *** | **-.274 **** | **-.243 **** | **.547 **** | **.521 **** | **.932 **** | **.818 **** | **.560 **** |  | **.981 **** | **.428 **** | **.361 **** | **.230 **** |
| **13-Area LAA ostium, mm²** | **.767 **** | **.543 **** | **.218 **** | **.155 *** | **-.271 **** | **-.237 **** | **.522 **** | **.528 **** | **.865 **** | **.896 **** | **.558 **** | **.986 **** |  | **.452 **** | **.367 **** | **.241 **** |
| **14-Depth of the LA, mm** | **.381 **** | **.731 **** | **.192 **** | **ns** | **-.213 **** | **ns** | **.235 **** | **.237 **** | **.310 **** | **.478 **** | **.296 **** | **.418 **** | **.450 **** |  | **.458 **** | **.419 **** |
| **15-Septum orifice distance, mm** | **.325 **** | **.728 **** | **ns** | **.179 *** | **-.208 *** | **ns** | **.144 *** | **.260 **** | **.306 **** | **.300 **** | **.296 **** | **.343 **** | **.345 **** | **.428 **** |  | **.562 **** |
| **16-TAOPLAW, cm²** | **.225 **** | **.673 **** | **.176 *** | **ns** | **-.156 *** | **ns** | **.141 *** | **ns** | **.219 **** | **.212 **** | **.150 *** | **.251 **** | **.254 **** | **.436 **** | **.547 **** |  |

This table shows all important correlation coefficients regarding CCTA measurement results. The values on the top represent the calculated correlation coefficients by Pearson, the values on the bottom by Spearman, respectively.

LAAV: left atrial appendage volume; LAV: left atrial volume; TAOPLAW: trapezoid area of the posterior left atrial wall;

*p<0.05; **p<0.01

**Figure Legends**

**Supplement Figure 1: Study population – selection criteria**

This flow chart explains the selection process of the study population. The top box shows the number of all patients included at the beginning. Each branching demonstrates one step of selection.

CCTA: cardiac computed tomography angiography

**Supplement Figure 2: Correlation of LA and LAA volumes**

The figure shows a linear regression model of LAA volume and LA volume. It demonstrates that per 10 mL increase of LA volume, LAA volume increases by 0.6 mL. The significance level of the model was p<0.001.

LAA: left atrial appendage; LA: left atrium

**Supplement Figure 3: Schematic demonstration of important LA and LAA measurements**

The picture in the back represents a three-dimensional left atrium in anterior view with important anatomical components. To improve the illustration of the measurements, a two-dimensional overlying scheme was added. The dotted lines and their respective numbers indicate the different measurements.

LAA: left atrial appendage; LA: left atrium; LSPV: left superior pulmonary vein; LIPV: left inferior pulmonary vein; RSPV: right superior pulmonary vein; RIPV: right inferior pulmonary vein; 1: LAA length; 2: distance to the first bend; 3: septum-orifice distance; 4: distance of the mitral valve annulus to the LA roof; 5: roof bottom line; 6: roof top line; 7: posterior wall box height
